# Surface-Displayed Amuc_1100 From *Akkermansia muciniphila* on *Lactococcus lactis* ZHY1 Improves Hepatic Steatosis and Intestinal Health in High-Fat-Fed Zebrafish

**DOI:** 10.3389/fnut.2021.726108

**Published:** 2021-10-13

**Authors:** Feng-Li Zhang, Ya-Lin Yang, Zhen Zhang, Yuan-Yuan Yao, Rui Xia, Chen-Chen Gao, Dong-Dong Du, Juan Hu, Chao Ran, Zhen Liu, Zhi-Gang Zhou

**Affiliations:** ^1^Sino-Norway Fish Gastrointestinal Microbiota Joint Lab, Institute of Feed Research of Chinese Academy of Agricultural Sciences, Beijing, China; ^2^Key Laboratory for Feed Biotechnology of the Ministry of Agriculture, Institute of Feed Research of Chinese Academy of Agricultural Sciences, Beijing, China; ^3^Hunan Provincial Key Laboratory of Nutrition and Quality Control of Aquatic Animals, Department of Biological and Environmental Engineering, Changsha University, Changsha, China

**Keywords:** Amuc_1100, *Lactococcus lactis*, fatty liver, intestinal health, microbiota, inflammation, zebrafish

## Abstract

Fatty liver and intestinal barrier damage were widespread in most farmed fish, which severely restrict the development of aquaculture. Therefore, there was an urgent need to develop green feed additives to maintain host liver and intestinal health. In this study, a probiotic pili-like protein, Amuc_1100 (AM protein), was anchored to the surface of *Lactococcus lactis* ZHY1, and the effects of the recombinant bacteria AM-ZHY1 on liver fat accumulation and intestinal health were evaluated. Zebrafish were fed a basal diet, high-fat diet, and high-fat diet with AM-ZHY1 (10^8^ cfu/g) or control bacteria ZHY1 for 4 weeks. Treatment with AM-ZHY1 significantly reduced hepatic steatosis in zebrafish. Quantitative PCR (*q*PCR) detection showed that the expression of the lipogenesis [peroxisome-proliferator-activated receptors (*PPAR*γ), sterol regulatory element-binding proteins-1c (*SREBP-1c*), fatty acid synthase *(FAS)*, and acetyl-CoA carboxylase 1 (*ACC1*)] and lipid transport genes (*CD36* and *FABP6*) in the liver were significantly downregulated (*p* < 0.05), indicating that AM-ZHY1 could reduce liver fat accumulation by inhibiting lipid synthesis and absorption. Moreover, supplementing AM-ZHY1 to a high-fat diet could significantly reduce serum aspartate aminotransferase (AST) and alanine aminotransferase (ALT) levels, indicating that liver injury caused by high-fat diets was improved. The expression of tumor necrosis factor (*TNF*)*-a* and interleukin (*IL*)*-6* in the liver decreased significantly (*p* < 0.05), while *IL-1*β and *IL-10* did not change significantly in the AM-ZHY1 group. Compared to the high-fat diet-fed group, the AM-ZHY1 group, but not the ZHY1 group, significantly increased the expression of intestinal tight junction (TJ) proteins (*TJP1a, claudina, claudin7, claudin7b, claudin11a, claudin12*, and *claudin15a*; *p* < 0.05). Compared to the high-fat diet group, the *Proteobacteria* and *Fusobacteria* were significantly reduced and increased in the AM-ZHY1 group, respectively. In conclusion, the recombinant bacteria AM-ZHY1 has the capacity to maintain intestinal health by protecting intestinal integrity and improving intestinal flora structure and improving fatty liver disease by inhibiting lipid synthesis and absorption. This study will lay a foundation for the application of AM protein in improving abnormal fat deposition and restoring the intestinal barrier in fish.

## Introduction

Fatty liver and intestinal barrier damage are widespread in most farmed fish, which severely restrict the development of aquaculture (1, 2). Therefore, there is an urgent need to develop green feed additives to maintain host liver and intestinal health. *Akkermansia muciniphila* is a human intestinal gram-negative anaerobic bacteria, with promising probiotic activities against many metabolic-related diseases such as obesity, diabetes, inflammatory bowel disease, etc. (3–7). Amuc_1100 (AM protein), a highly abundant pili-like membrane protein of *A. muciniphila*, partly recapitulates the beneficial effects of *A. muciniphila* (8, 9). Furthermore, AM protein can modulate host immune responses and specifically induce high cytokine production in peripheral blood mononuclear cells (PBMCs) likely *via* Toll-like receptor 2 (TLR2) signaling (10). Oral AM protein can activate the immune response of mice potentially *via* TLR2 (8). Other than that, AM protein can restore gut barriers and enhance the transepithelial resistance of Caco-2 cells (10). Specifically, oral AM protein enhanced the mouse intestinal barrier likely by acting on TLR2 and restoring the appropriate expression of tight junction proteins (8). AM protein can also alleviate metabolic endotoxemia, improve glucose and lipid metabolism, and reduce fat mass in high-fat diet-fed mice (8). These results indicate that *A. muciniphila* and AM protein have potential as a green feed additive in aquaculture.

As a strictly anaerobic bacteria, *A. muciniphila* is difficult to produce at an industrial scale, which limits its application in aquaculture. Compared with the bacteria, its probiotic element, AM protein, is easy to express heterologously and can be produced on a large scale, with great application potential. The applications of protein as a feed additive will face a series of problems such as a complex expression and purification process, poor stability, poor resistance, and high cost. A microbial [especially generally recognized as safe (GRAS) *lactic acid bacteria*] surface display can well circumvent the above problems, becoming attractive platforms for the surface display of heterologous proteins in various fields.

In this study, AM protein was anchored to the cell wall of fish-derived *Lactococcus lactis* ZHY1 using surface display technology. The lipid-lowering effect of recombinant bacteria on hepatic steatosis caused by a high-fat diet was tested, and the effects of the recombinant bacteria on intestinal barrier function and the inflammatory response of zebrafish were evaluated. This study will lay a foundation for the application of AM protein in improving abnormal fat deposition and restoring the intestinal barrier in fish.

## Materials and Methods

### Bacterial Strains and Growth Conditions

In this experiment, *L. lactis* was isolated from the intestine of *Acipenser sinensis*. After the intestinal contents were suspended in 0.1 mM of phosphate-buffered saline (PBS), and then spread on a De Man, Rogosa and Sharpe (MRS) agar medium supplemented with calcium carbonate. Due to its high yield, we chose it as the carrier for the surface display, and it was identified as *L. lactis* by 16s rRNA sequencing, named ZHY1 (preservation number: CGMCC No. 22024). This strain was grown in a medium in M17 broth containing 0.5% glucose (HB0391, Qingdao Hope Bio-Technology Co., Ltd., Qingdao, China) and was cultured statically at 30°C.

### Construction and Expression of Recombinant Bacteria AM-ZHY1

**Figure 1:**
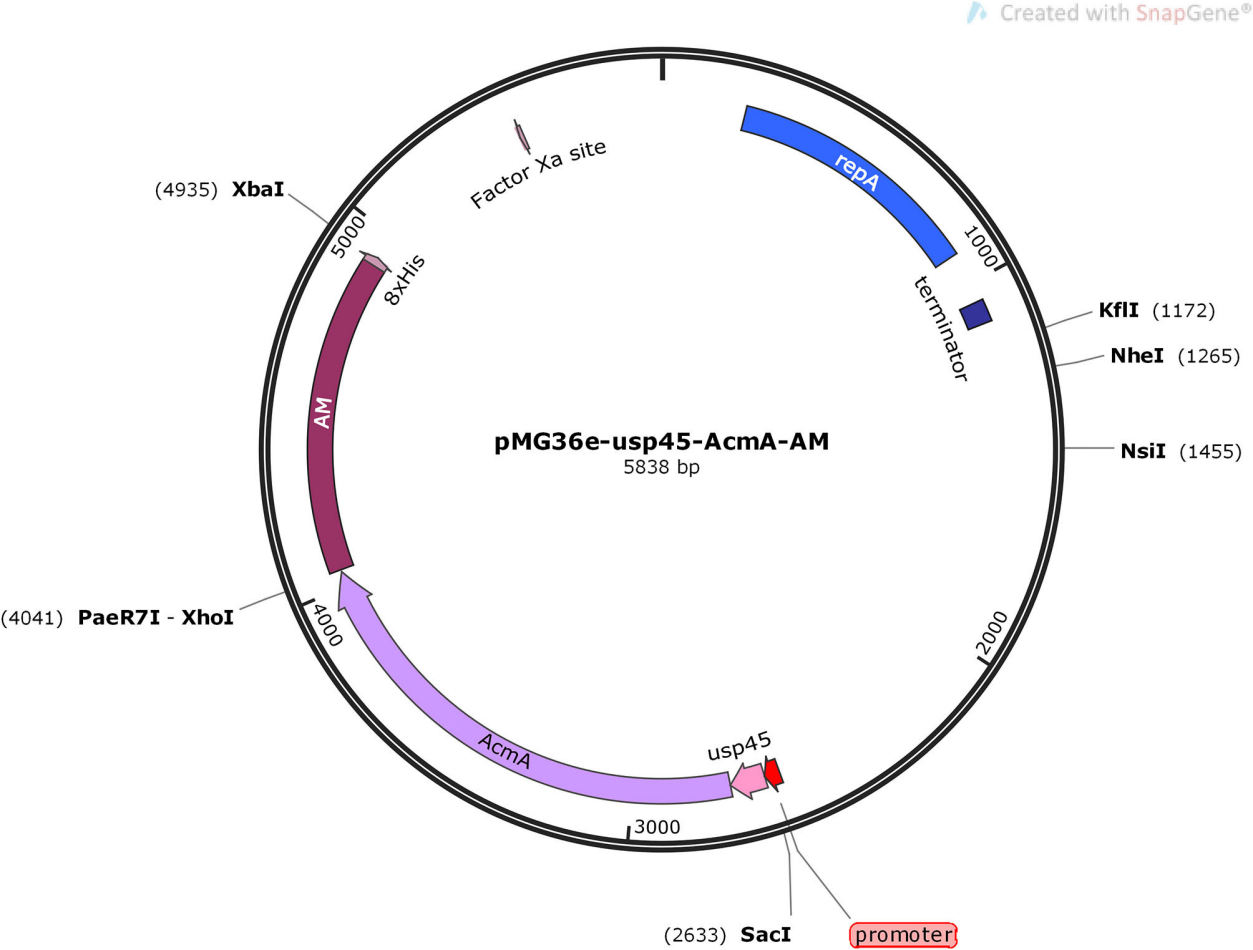
Schematic view of building the pMG36e-usp45-AcmA-AM plasmid.

The 900-bp nucleotide coding sequence of AM was synthesized and cloned into the pET28a plasmid (Generay Biotech, Shanghai, China) and digested with XhoI (R0146S, NEB, Beijing, China) and XbaI (R0145V, NEB). The gene fragment of usp45 and AcmA was amplified by fusion PCR from the signal peptide genomic major lactococcal autolysin N-acetylmuramidase DNA of *L. lactis* MG1363 using a set of primers, usp45F (5′-CGAGCTCATATGAAAAAAAAGATTA-3′) and AcmAR (5′-GTCAGTATCTGCGAATAAAACTCGAG-3′). The amplification reaction was performed using the Q5 High-Fidelity DNA Polymerase following the recommendations of the manufacturer, and the amplicon was digested by SacI (R0156S, NEB) and XhoI. The two M0515, NEB digested products mentioned above were inserted into the shuttle vector pMG36e, and the ligation mixture was transformed into *Escherichia coli* MC1061 (TIANGEN, Beijing, China). The correct recombinant plasmid, pMG36e-usp45-AcmA-AM, was identified by DNA sequencing (Sangon Biotech, Shanghai, China) (Figure 1).

*Lactococcus lactis* ZHY1 competent cells were prepared and transformed as described previously with slight modifications (11). The overnight culture of *L. lactis* was inoculated at a 5% ratio into a fresh GM17 medium with 2.5% glycine, and then cultivated until OD_600_ = 0.5. The bacteria were washed two times with a pre-cooled washing buffer (17.1% sucrose, 1% glycerol), and then resuspended in a pre-chilled washing buffer and mixed with the recombinant plasmid pMG36e-usp45-AcmA-AM. The electroporator was pulsed, and the cells were plated on a GM17 agar medium containing 10 ug/ml of erythromycin. The recombinant bacteria AM-ZHY1 were selected after 48 h of culture by PCR (MG36eF: 5′-ACTCTCTGGGGACTTTCG-3′; MG36eR: 5′-TCGCCTTTACCAACTGTC-3′).

### Sodium Dodecyl Sulfate-Polyacrylamide Gel Electrophoresis (**SDS-PAGE**) and Western Blot

The inoculation of AM-ZHY1 was performed into a GM17 medium containing 10 μg/ml erythromycin and cultured at 30°C for 48 h. The culture cells were then collected and resuspended in 1 ml of Tris-HCl (50 mM, pH 7), which contained 10 mM of magnesium chloride (MgCl_2_) and 4% SDS buffer, at 37°C for 2 h. The mixture was denatured by heating at 100°C before loading, and the supernatant was the lysed cell wall components. The samples were analyzed by 12% SDS-PAGE (BioRad, Hercules, CA, USA) and electrophoresis at a 120-V constant voltage for 2 h.

After electrophoresis, the protein bands were transferred to a polyvinylidene fluoride (PVDF) membrane (0.45 uM, IPVH00010, Millipore, Beijing, China). After the electrotransfer was completed, the membrane was blocked in 5% skim milk and incubated overnight at 4°C with an anti-His tag monoclonal antibody (1:1,000 dilution, AB102, TIANGEN). Then, Goat Anti-mouse IgG, HRP Conjugated (1:200 dilution, CW0102, cwbiotech, Beijing, China) was added at room temperature for 2 h after washing with 0.05% TBST (50 mM Tris-HCl, 150 mM NaCl, 0.05% Tween 20). Chemiluminescent HRP Substrate (WBKLS0100, Millipore) was used to visualize protein bands.

### Diets Preparation and Fish Husbandry

The experiment used zebrafish (1-month-old), AB strain. The zebrafish (n = 480, mean initial weight: 50 mg) were randomly allocated to 20 3-L tanks in the recirculation system. The zebrafish were fed for 4 weeks at 26°C, 12/12 light/dark cycle, dissolved oxygen ≥6 mg/L, total ammonia content ≤0.02 mg nitrogen/L, pH 7. All experimental and animal care procedures were approved by the Feed Research Institute of the Chinese Academy of Agricultural Sciences Animal Care Committee, under the auspices of the China Council for Animal Care (Assurance No. 2018-AF- FRI-CAAS-001).

The feed ingredients and formulas of the basal diet and high-fat diet are shown in Table 1. The recombinant bacteria, AM-ZHY1, and the control bacteria, ZHY1, were added to the high-fat diet at the amount of 10^8^ cfu/g before being named the AM-ZHY1 group and ZHY1 group, respectively. Before preparation and after the feed was crushed (stored at −20°C), the viable cell numbers of *L. lactis* were determined by the plate counting method. The zebrafish were fed with the experimental diets two times a day (9:00, 17:00) at a ratio of 6% of body weight.

**Table 1:** Ingredient and nutrient composition of the basal diet and high-fat diet.

**Ingredients (g/kg diet)**	**basal diet**	**High-fat diet**
Casein	400	400
gelatin	100	100
dextrin	280	160
Lard oil	0	80
Bean oil	60	80
Lysine	3.3	3.3
VC lecithin	1	1
Vitamin premix^a^	2	2
Mineral premix^b^	2	2
Calcium dihydrogen phosphate	20	20
Choline chloride	2	2
Sodium alginate	20	20
Microcrystalline cellulose	40	40
Zeolite powder	69.7	89.7
Total	1,000	1,000
Proximate composition (g/kg dry diet)		
Crude protein	449.4	448.9
Crude lipid	53.9	157.0

*Vitamin premix^a^ and mineral premix^b^ were obtained from Beijing Xinlu United Aquatic Products Co., Ltd. The nutrition provided by the feed meets NRC standards*.

### Growth Performance

After 4 weeks of feeding, each fish in the tanks was weighed to assess body weight gain (WG), feed conversion ratio (FCR), and survival rate (SR) (12), which showed the following: WG: [100 × (W_t_ – W_0_)/W_0_], FCR: (W_f_/W_d_) × 100; SR = (N_0_/N_t_) × 100; where W_0_ (g), W_t_ (g), W_f_, W_d_, N_0_, and N_t_ are the initial weight, final weight, total food intake, total weight gain, initial quantity, and final quantity of the zebrafish, respectively.

### Liver TAG Content and HE Staining

The determination of the triacylglycerol (TAG) content of the zebrafish livers mainly referred to the method of Zhang et al. (13). The liver tissues (5–10 mg) from five zebrafish were homogenized in a PBS buffer. About 5 ml of methanol/chloroform (1:2) solution was added to the homogenate, and it was centrifuged (3,000 rpm, 5 min) to avoid protein sticking to the wall of the centrifuge tube. The organic components were carefully dried at 70°C with nitrogen steam. Then, the dried lipids were emulsified in chloroform with 1% Triton X-100. Each of the dried emulsified lipids was dissolved in deionized water, and we then detected the amount of TAG contained in the unit weight of the sample using a triglyceride reagent (T2449, Sigma-Aldrich, Saint Louis, MO, USA) and a free glycerol reagent (F6428, Sigma-Aldrich).

The zebrafish liver was carefully removed and immediately fixed in 4% paraformaldehyde. After embedding in paraffin, the sections were cut and stained with hematoxylin-eosin (HE). The cellular lipid vacuoles of the liver were observed under a microscope (Leica DMIL-LED, Wetzlar, Germany).

### Serum Biochemical Measurements

Blood samples were collected from the zebrafish as previously described (14). The activities of zebrafish serum alanine aminotransferase (ALT) and aspartate aminotransferase (AST) were detected using commercial diagnostic kits (Nanjing Jiancheng Bioengineering Institute, China) according to the instructions of the manufacturer. Serum ALT and AST activities were expressed as enzyme activity units per liter (U/L).

### Quantitative Real-Time PCR Analysis

The total RNA of the zebrafish liver and intestine samples were isolated using a Trizol reagent (Invitrogen, Waltham, MA, USA) and reversed transcribed to cDNA using a FastKing-RT Supermix (KR118, TIANGEN). The expression of *CD36, FABP6*, sterol regulatory element-binding proteins-1c (*SREBP-1c*), peroxisome-proliferator-activated receptors *(PPAR*γ), acetyl-CoA carboxylase 1 (*ACC1)*, adipose triglyceride lipase (*ATGL*), and uncoupling protein 2 (*UCP2*) of the liver; tumor necrosis factor (*TNF*)*-*α, interleukin (*IL*)*-6, IL-10*, and *IL-1*β of the liver; and the intestinal tight junction (TJ) proteins (*TJP1a, claudina, claudin7, claudin7b, claudin11a, claudin12*, and *claudin15a*) were determined by quantitative real-time PCR (*q*RT-PCR) using the SYBR Green Supermix (FP204, TIANGEN) on a Light Cycler 480 system (Roche, Basel, Switzerland). The results were analyzed by the 2^−ΔΔ^Ct method (15). The primers of all detected genes are listed in Tables 2, 3, and the reference gene was Rps11.

**Table 2:** The primer sequences for quantitative (*q*PCR) analysis of lipid metabolism and inflammation-related genes.

**Gene name**	**Forward primer (5′-3′)**	**Reverse primer (5′-3′)**
*Rps11*	ACAGAAATGCCCCTTCACTG	GCCTCTTCTCAAAACGGTTG
*(Reference*		
*gene)*		
*CD36*	TGAACAAAATCAAGGAGC	ATCCGGGAAATCAGCTCATTCTT
	ACACAA	
*FABP6*	ACCCACAACCATCATCTC	TCTTTACCGTCCCATTTC
*SREBP-1c*	CAGAGGGTGGGCATGCTGGG	ATGTGACGGTGGTGCCGCTG
*ATGL*	GCGTGACGGATGGAGAAA	AGGCCACAGTAAACAGGAATAT
*FAS*	GGAGCAGGCTGCCTCTGTGC	TTGCGGCCTGTCCCACTCCT
*PPARγ*	CCTGTCCGGGAAGACCAGCG	GTGCTCGTGGAGCGGCATGT
*ACC1*	GCGTGGCCGAACAATGGCAG	GCAGGTCCAGCTTCCCTGCG
*UCP2*	TGCCACCGTGAAGTTTATTG	CCTCGATATTTCACCGGACC
*TNFα*	AAGGAGAGTTGCCTTTACCG	ATTGCCCTGGGTCTTATGG
*IL-6*	TCAACTTCTCCAGCGTGATG	TCTTTCCCTCTTTTCCTCCTG
*IL-10*	TCACGTCATGAACGAGATCC	CCTCTTGCATTTCACCATATCC
*IL-1β*	GGCTGTGTGTTTGGGAATCT	TGATAAACCAACCGGGACA

**Table 3:** Primers sequences for *q*PCR analysis of tight junction proteins.

**Gene name**	**Forward primer (5′-3′)**	**Reverse primer (5′-3′)**
*Rps11 (Reference gene)*	ACAGAAATGCCCCTTCACTG	GCCTCTTCTCAAAACGGTTG
*Claudin7*	GGAGCCCTCTGTAGCATTGTT	ATTAGGAGATGACTGACCCTTTGA
*Claudin11a*	ACCAGAAAGTGCCAAGAACA	AAAGCCAAAGGACATCAGACC
*Claudin12*	CCTCCGTCTGGTTCCTCT	GCTTTGGGTCATTGTGGG
*Claudin7b*	GGAGCCCTCTGTAGCATTGTTG	ATTAGGAGATGACTGACCCTTTGA
	TGTTG	
*Claudin15a*	CTCTGCTCGCTCTATCTGGTTA	TTTCACGGGTGGGATGGTAT
*TJP1a*	CAAAGACCAACAGCACTGCC	GTGGTTTAGCGGTGATGGGA
*Claudina*	TCAGTGAGTTTCCTCCTATTGT	TATTCGCCGCTCCTCTTTT
*TJP1b*	CCCACAGATCTCCCCAAACC	CTGGAGCTCTGTAGGAGGGT

### The Influence of Gut Microbes

After 4 weeks of feeding, the intestinal contents of the zebrafish from the four diet groups were obtained 4 h after the last feeding. The intestinal sample of each group contained six replicates, and six fish were pooled as a replicate. Afterward, DNA was extracted with a Fast DNA spin kit (MP, Biomedicals, Solon, OH, USA), according to the instructions of the manufacturer. The V3–V4 region of 16S rRNA was amplified with U341F (5′-CGGCAACGAGCGCAACCC-3′) and U806R (5′-CCATTGTAGCACGTGTGTAGCC-3′). The high-throughput sequencing of the gut microbes was performed at the MajorBio (Shanghai), using the Illumina HiSeq platform. Microbiota sequencing data of zebrafish intestinal flora are available from NCBI Sequence Read Archive with accession number PRJNA739824. The UPARSE-operational taxonomic unit (OTU) algorithm was used to control the quality of raw pair-end readings (16). The RDP classifier Bayesian algorithm was used to perform a taxonomic analysis at the 97% similar level (17). According to the results of the taxonomic analysis, a principal component analysis of different groups was performed using R 3.3.1 at (18).

### Statistical Analysis

The statistical data were analyzed by the GraphPad Prism 5 software (GraphPad Software Inc., San Diego, CA, USA). All statistics were from six repetitions. Differences among groups were assessed using Student's *t*-test. *p* < 0.05, *p* < 0.01 and *p* < 0.001 were considered significant differences.

## Result

### Oral Administration of AM-ZHY1 Reduces Weight Gain and Liver Lipid Deposition

**Figure 2:**
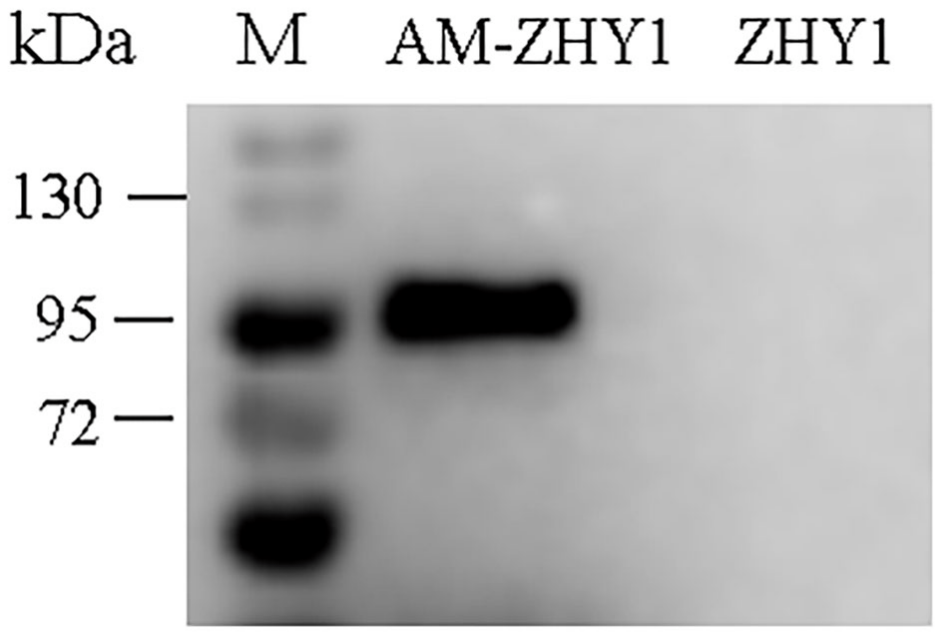
Western blotting detects the expression of Amuc_1100 (AM) on ZHY1. Line 1: protein marker (26630, Thermo Scientific), line 2: the whole cell wall protein of recombinant strain AM-ZHY1, line 3: the whole cell wall protein of control strain ZHY1.

**Figure 3:**
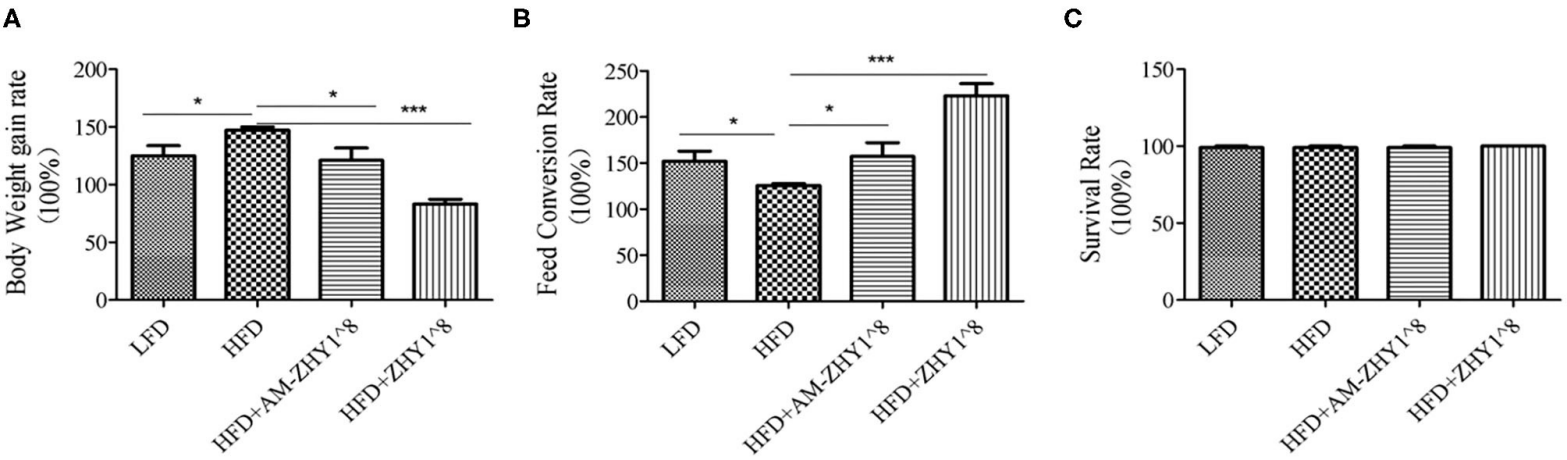
Effects of different diets on the **(A)** body weight gain, **(B)** feed conversion rate, and **(C)** survival rate of zebrafish. Data represent as the means ± SEM. The asterisks on the horizontal line represent significant differences between the two groups. *p < 0.05, ***p < 0.001.

As shown in Figure 2, the fusion protein AcmA-AM was successfully expressed in the surface of the recombinant bacteria AM-ZHY1. After 4 weeks of the feeding experiment, the WG, FCR, and SR of the four groups of zebrafish were calculated. As shown in Figure 3, both the WG of the AM-ZHY1 and ZHY1 groups showed 17.7 and 43.5% lower than high-fat diet group. The FCR of the AM-ZHY1 group was 25% higher than the high-fat diet group. The SRs was unaffected by the dietary treatments.

**Figure 4:**
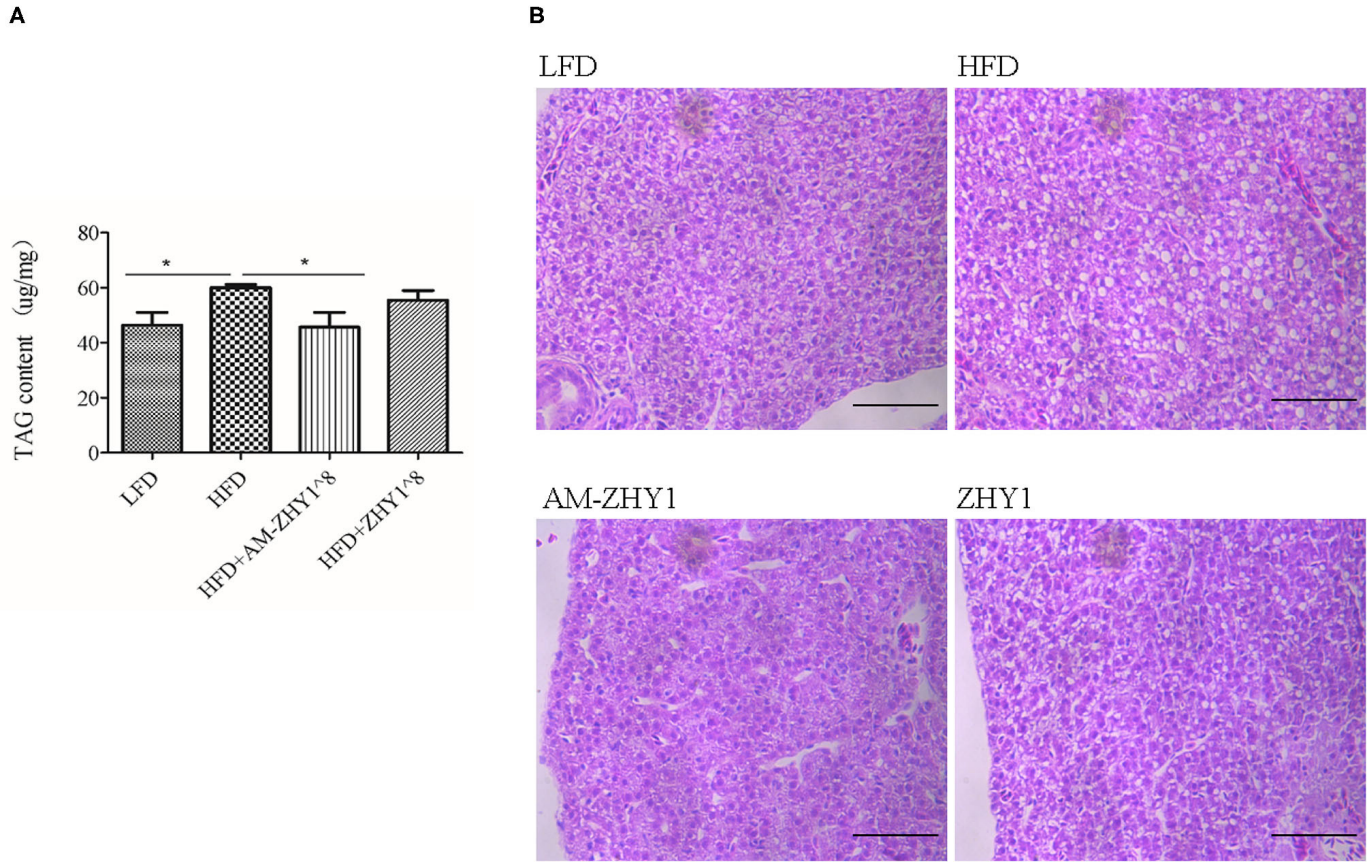
Triacylglycerol (TAG) content of the livers of zebrafish in different groups. **(A)** TAG content of the liver, the significance level was determined by a Student's *t*-test (*p < 0.05). **(B)** Histological changes of liver sections measured by HE staining (scale bar is 50 μm).

**Figure 5:**
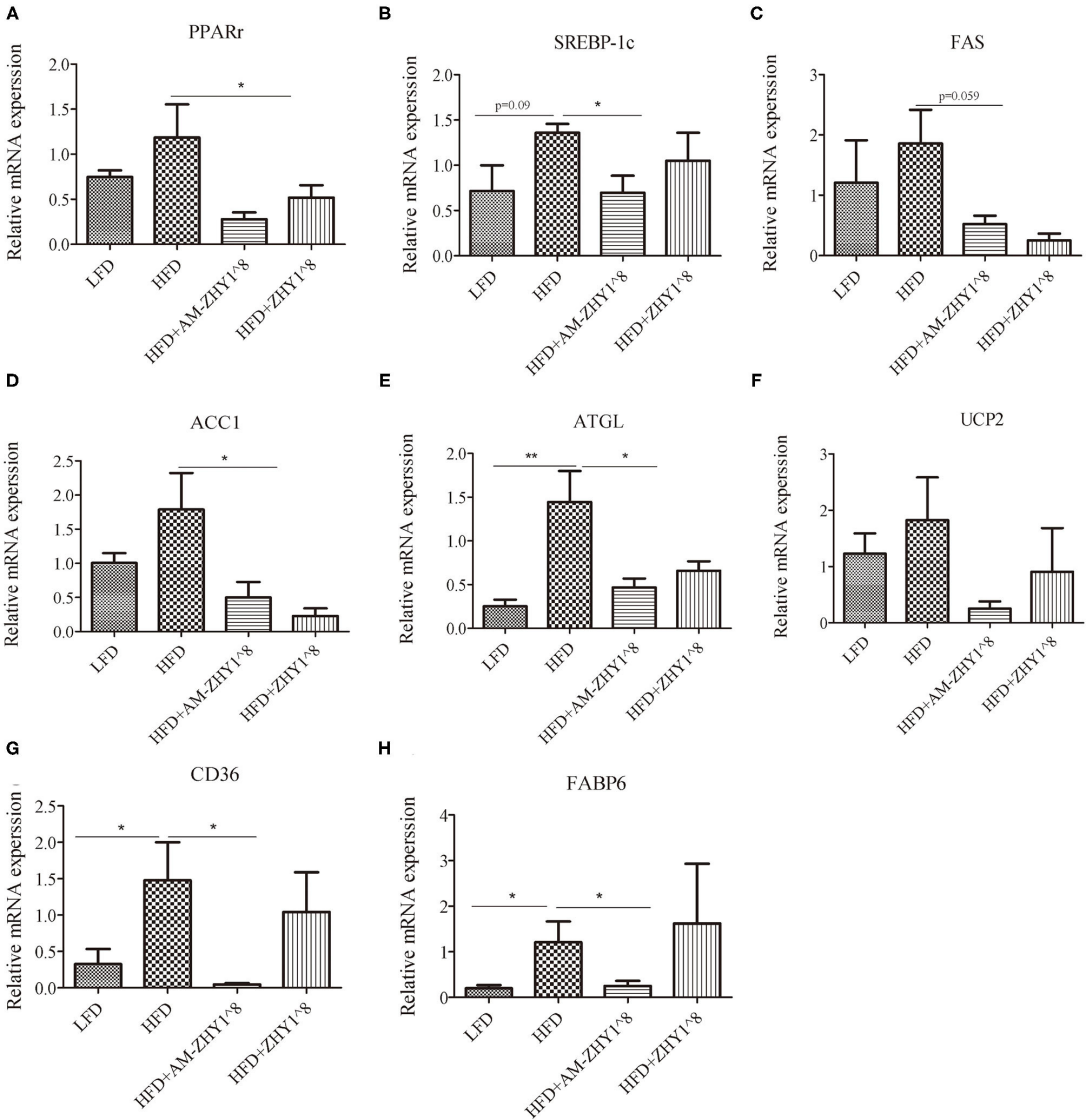
Relative mRNA expression of lipid metabolic-related genes PPARγ **(A)**, SREBP-1c **(B)**, FAS **(C)**, ACC1 **(D)**, ATGL **(E)**, UCP2 **(F)**, CD36 **(G)**, FABP6 **(H)** of the livers of zebrafish in different groups. Data are presented as mean ± SEM (*n* = 6). The asterisks on the horizontal line represent significant differences between the two groups. *p < 0.05, **p < 0.01.

The improvement of AM-ZHY1 on hepatic steatosis induced by the high-fat diet is shown in Figures 4A,B. The high-fat diet significantly increased liver fat accumulation compared with the basal diet (29.5% higher; *p* < 0.05). The supplementation of AM-ZHY1, but not ZHY1, significantly reduced TAG content in the liver compared with the high-fat diet group (by 31.6 and 7.6%, respectively; *p* < 0.05; *p* = 0.28). Moreover, the TAG content in zebrafish fed the high-fat diet with AM-ZHY1 was 26% lower than in the fish fed the high-fat diet with ZHY1 (*p* < 0.05). Accordingly, histopathologic analysis of the HE-stained liver sections showed hepatic steatosis in zebrafish fed high-fat diets, which was obviously reversed by the addition of AM-ZHY1. The effect of AM-ZHY1 on the lipid metabolism of the high-fat diet-fed zebrafish was investigated (Figure 5). The process of lipid metabolism mainly includes fat synthesis genes, lipolysis genes, and fat absorption-related genes at the genetic level. The expression of key enzymes involved in the *de novo* fatty acid synthesis pathway, including *ACC1* and fatty acid synthase (*FAS*), was obviously lower in the AM-ZHY1 group than the high-fat diet group (by 71.9 and 71.9%, respectively; *p* < 0.05; *p* < 0.05). The mRNA expression of the transcription factors that regulate fatty acid and triglyceride synthesis, including *PPAR*γ and *SREBP-1c*, was significantly downregulated in the AM-ZHY1 group (*p* < 0.05; *p* < 0.05). However, the expression of *PPAR*γ and *SREBP-1c* was not significantly reduced in zebrafish fed with ZHY1 compared with those fed the high-fat diet (*p* = 0.12; *p* = 0.42). The AM-ZHY1 significantly inhibited the expression of the lipid absorption genes *CD36* and *FABP6* (*p* < 0.05; *p* < 0.05). However, the expression of these two genes in zebrafish fed with the high-fat diet supplemented with control bacteria ZHY1 did not decrease significantly (*p* = 0.58; *p* = 0.74). The addition of AM-ZHY1 and ZHY1 to the high-fat diet both inhibited the expression of the *ATGL* of zebrafish (*p* < 0.05; *p* = 0.07). Taken together, these results demonstrated that the supplement AM-ZHY1 with the high-fat diet led to the improvement of hepatic steatosis. Furthermore, this regulatory effect of AM-ZHY1 on liver lipid accumulation was through inhibiting lipid synthesis and absorption, rather than promoting lipolysis.

### AM-ZHY1 Relieves High-Fat Diet-Induced Liver Injury by Regulating Lipid Metabolism and Inflammatory Responses

**Figure 6:**
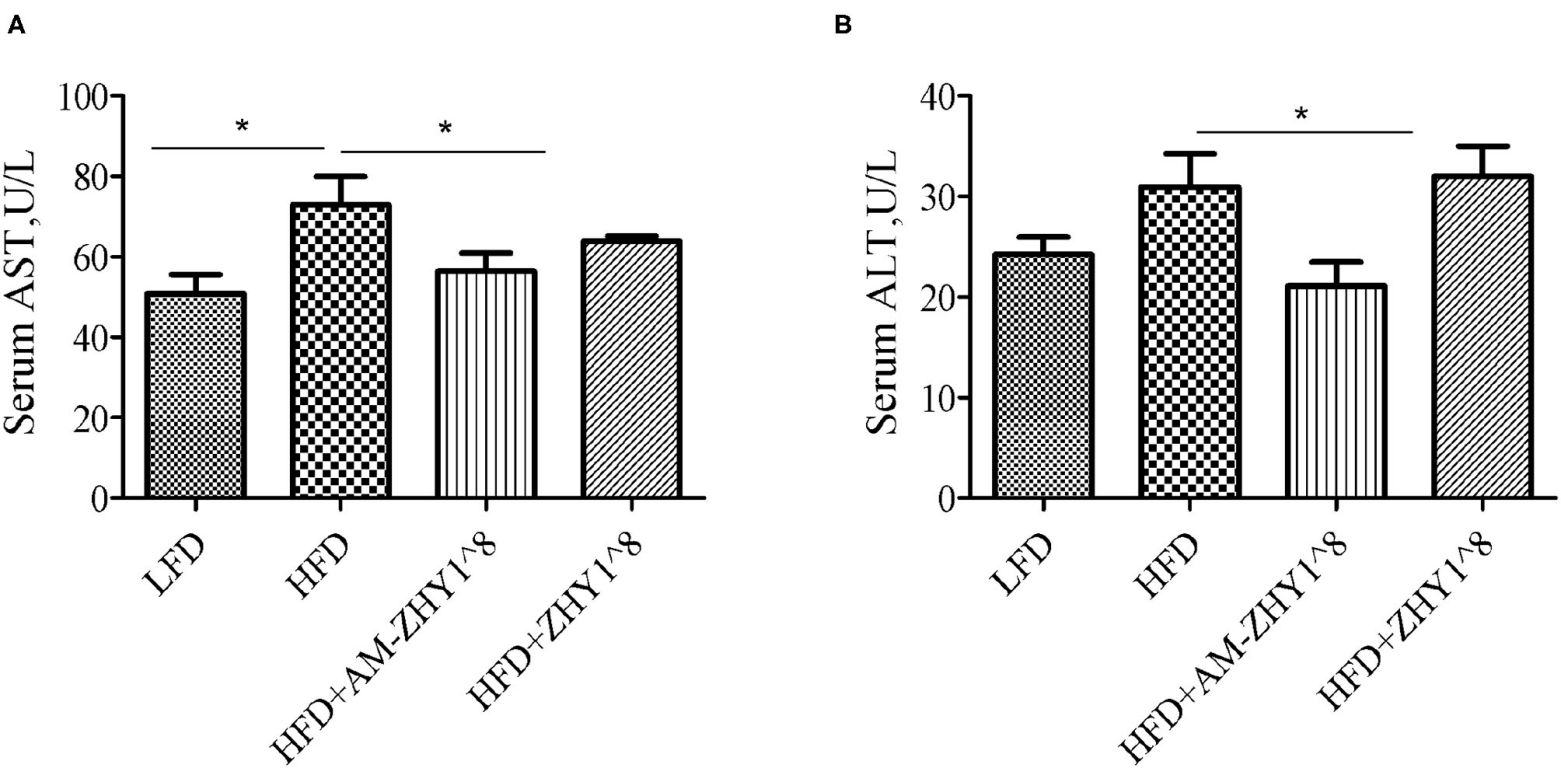
Serum alanine aspartate aminotransferase [AST, **(A)**] and alanine aminotransferase [ALT, **(B)**] levels of zebrafish fed with a basal diet and high-fat diet, with AM-ZHY1(10^8^cfu/g) and ZHY1(10^8^cfu/g) supplemented with the high-fat diet. Data are presented as mean ± SEM (*n* = 6). The asterisks on the horizontal line represent significant differences between the two groups. *p < 0.05.

**Figure 7:**
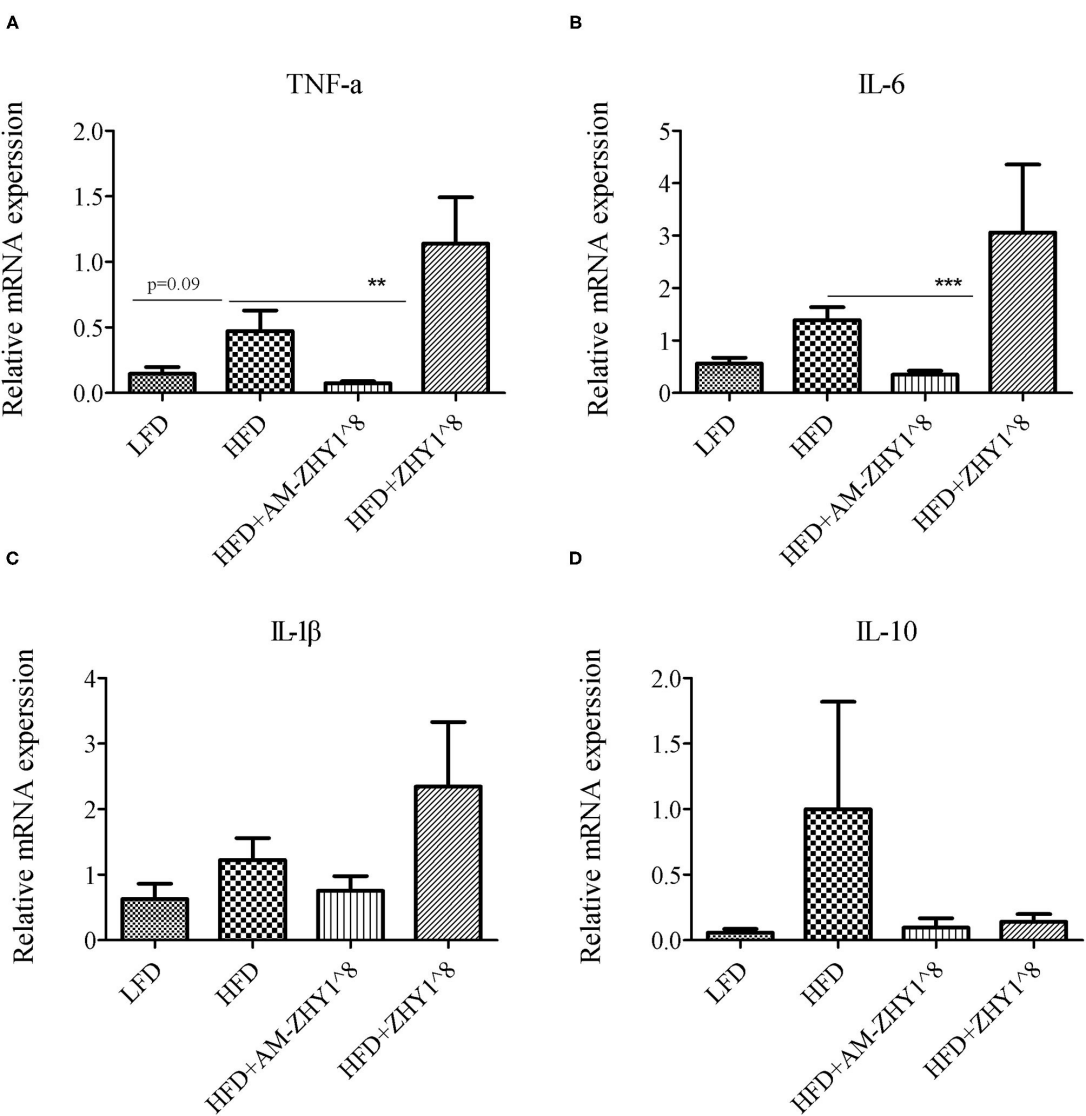
Relative mRNA expression of inflammation related genes: TNFα **(A)**, IL-6 **(B)**, IL-1β **(C)**, IL-10 **(D)** of liver of zebrafish of different groups. Data are presented as mean ± SEM (*n* = 6). The asterisks on the horizontal line represent significant differences between the two groups. **p < 0.01, ***p < 0.001.

After the feeding experiment, the zebrafish serum ALT, AST, and liver inflammatory factor expression levels were tested to examine the effect of AM-ZHY1 on liver health. Compared with the high-fat diet group, the levels of ALT and AST were significantly reduced in the AM-ZHY1 group (*p* < 0.05, *p* < 0.05; Figure 6), and the expression level of *TNF-a* and *IL-6* of the liver were significantly decreased in the AM-ZHY1 group (*p* < 0.05, *p* < 0.05; Figures 7A,B). The expression level of *IL-1*β and *IL-10* had no significant changes in the AM-ZHY1 group (*p* = 0.24, *p* = 0.18; Figures 7C,D). These results indicated that an AM-ZHY1 treatment could alleviate liver injury and the inflammation caused by a high-fat diet.

### AM-ZHY1 Improves High-Fat Diet-Induced Intestinal Injury by Altering Gut Microbiota Dysbiosis and Protecting Intestinal Integrity

**Figure 8:**
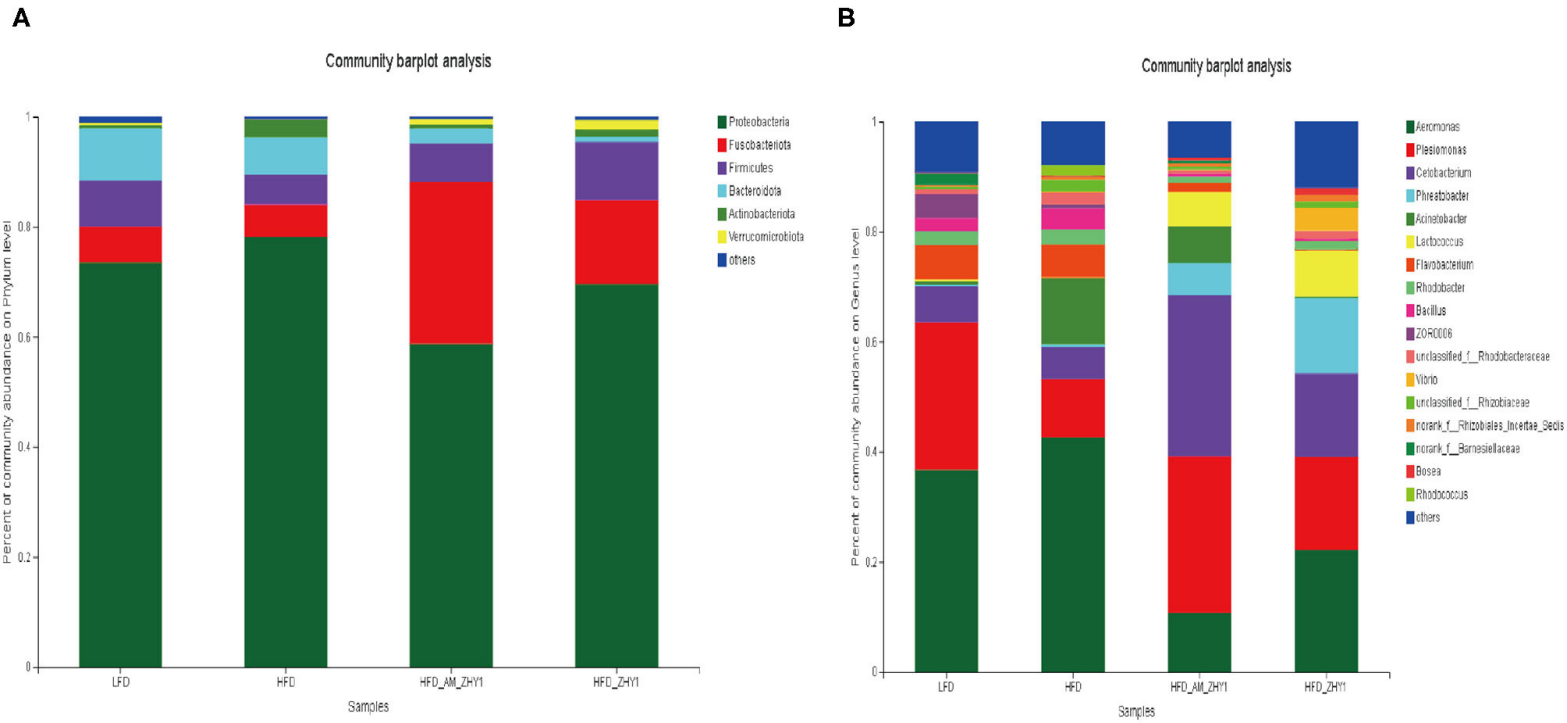
The effect of different diets on the relative abundance of intestinal flora at the phylum in zebrafish **(A)** and the genus **(B)** level. LFD, basal diet; HFD, high-fat diet; HFD_AM_ZHY1, supplement 10^8^cfu/g AM-ZHY1 based on a high-fat diet; HFD_ZHY1, supplement 10^8^cfu/g ZHY1 based on a high-fat diet.

A total of 1,519,923 high-quality 16S rRNA sequences were generated from 30 samples. After subsampling each sample to an equal sequencing depth of 33,004 reads per sample, sequences were clustered into 1,706 OTUs. Treating with AM-ZHY1 could significantly change the structure of zebrafish intestinal microbiota at both the genus and phylum level (Figure 8). Compared to the high-fat diet group, Proteobacteria were significantly reduced, and Fusobacteria were significantly increased in the AM-ZHY1 group (*p* < 0.05; *p* < 0.05; Table 4). At the genus level, the relative content of *Aeromonas* was significantly reduced in the AM-ZHY1 group compared with the high-fat diet group (*p* < 0.05). The relative abundance of *Cetobacterium* was significantly increased in the AM-ZHY1 group (*p* < 0.05), which is higher than any other group. The relative content of *Plesiomonas* in the AM-ZHY1 group was consistent with that in the basal diet group. The relative contents of *Acinetobacter* and *Bacillus* did not change significantly among these groups (Table 5).

**Table 4:** The relative abundance of each group of intestinal flora at the phylum level.

	**LFD**	**HFD**	**HFD_AM-ZHY1**	**HFD_ZHY1**
*Proteobacteria*	73.51 ± 11.90^ab^	78.12 ± 6.56^b^	58.74 ± 16.23^a^	69.55 ± 9.57^ab^
*Fusobacteriota*	6.57 ± 4.16^a^	5.89 ± 1.78^a^	29.33 ± 16.03^b^	15.25 ± 11.33^ab^
*Firmicutes*	8.35 ± 2.57^a^	5.48 ± 3.13^a^	7.11 ± 2.82^a^	10.60 ± 7.82^a^
*Bacteroidota*	9.46 ± 6.15^a^	6.78 ± 4.65^ab^	2.70 ± 2.22^bc^	1.01 ± 0.86^c^
*Actinobacteriota*	0.61 ± 0.26^a^	3.13 ± 2.32^b^	0.719 ± 0.37^a^	1.20 ± 0.84^a^
*Verrucomicrobiota*	0.30 ± 0.19^a^	0.096 ± 0.034^a^	0.97 ± 0.66^a^	1.77 ± 2.26^a^

*Values are means ± SEMs; n = 6 (pool of 3 zebrafish per sample). In the same line, different letters represent significant differences, p < 0.05*.

*LFD, basal diet; HFD, high-fat diet; HFD_AM-ZHY1, supplement 10^8^cfu/g AM-ZHY1 based on a high-fat diet; HFD_ZHY1, supplement 10^8^cfu/g ZHY1 based on a high-fat diet*.

**Table 5:** The relative abundance of each group of intestinal flora at the genus level.

	**LFD**	**HFD**	**HFD_AM-ZHY1**	**HFD_ZHY1**
*Aeromonas*	36.69 ± 21.23^bc^	42.58 ± 23.57^c^	10.76 ± 5.29^ab^	22.05 ± 21.15^abc^
*Plesiomonas*	24.25 ± 10.79^a^	10.63 ± 9.07^a^	29.42 ± 10.36^a^	17.09 ± 16.95^a^
*Cetobacterium*	6.56 ± 4.16^a^	5.88 ± 1.78^a^	29.33 ± 16.03^b^	15.24 ± 11.32^ab^
*Acinetobacter*	0.66 ± 0.32^a^	12.04 ± 5.49^a^	6.63 ± 6.15^a^	0.35 ± 0.33^a^
*Lactococcus*	0.25 ± 0.09^a^	0.09 ± 0.05^a^	6.27 ± 2.66^b^	8.33 ± 5.94^b^
*Bacillus*	2.38 ± 1.54^ab^	3.81 ± 2.67^a^	0.54 ± 0.28^a^	0.33 ± 0.24^a^

*Values are means ± SEMs; n = 6 (pool of 3 zebrafish per sample). In the same line, different letters represent significant differences, p < 0.05*.

*LFD, basal diet; HFD, high-fat diet; HFD_AM-ZHY1, supplement 10^8^cfu/g AM-ZHY1 based on a high-fat diet; HFD_ZHY1, supplement 10^8^cfu/g ZHY1 based on a high-fat diet*.

**Figure 9:**
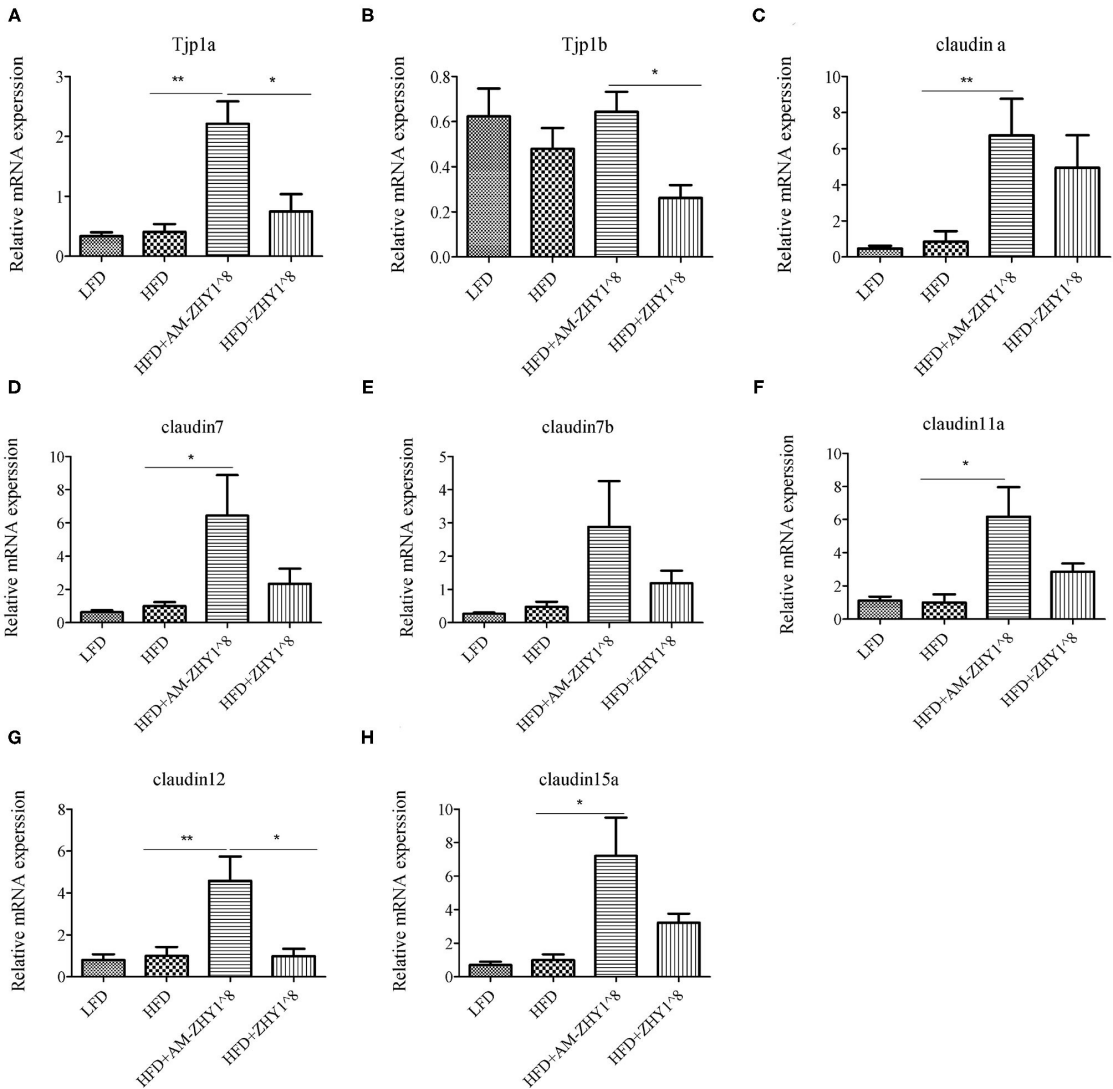
Relative mRNA expression of the tight junction protein genes TJP1a **(A)**, TJP1b **(B)**, Claudina **(C)**, Claudin7 **(D)**, Claudin7b **(E)**, Claudin11a **(F)**, Claudin12 **(G)**, Claudin15a **(H)** of the intestines of zebrafish in different groups. Data are presented as mean ± SEM (*n* = 6). The asterisks on the horizontal line represent significant differences between the two groups. (*p < 0.05,**p < 0.01).

The effect of AM-ZHY1 supplementation on intestinal health was elucidated by detecting the gene expression of *TJP1a, claudina, claudin7, claudin7b, claudin11a, claudin12*, and *claudin15a*. The AM-ZHY1 group, but not the ZHY1 group, significantly increased the expression of the above-mentioned intestinal TJ proteins vs. the high-fat diet group (*p* < 0.05; Figure 9). The above results indicated that AM-ZHY1 could improve zebrafish intestinal barrier structure.

## Discussion

In this experiment, the AM protein was displayed on the surface of *L. lactis* ZHY1, and its probiotic effects, including relieving high-fat diet-induced liver steatosis and improving the barrier function of the intestinal tract, were confirmed in zebrafish. For the first time, AM protein was used to treat fish fatty liver in aquaculture, and this research, combined with surface display technology, advanced its practical application.

Studies have shown that AM protein could decrease the body weight and fat content of high-fat diet-fed mice (8). In this experiment, AM-ZHY1 reduced the high-fat diet-induced weight gain to the basal diet level, similar to the same function in mammals. However, the ZHY1 group could also significantly reduce weight gain. This may be due to the fact that *lactic acid bacteria* themselves could play an anti-obesity effect by regulating the lipid metabolism of the body and changing the composition of intestinal microbes (9, 19–22). Next, we tested the content of liver TAG and found that the content of liver TAG in the ZHY1 group did not decrease compared with the high-fat diet group, but the AM-ZHY1 group significantly reduced the accumulation of liver fat. This further confirmed that AM protein-induced weight loss by reducing liver fat accumulation, while ZHY1-induced weight loss may be due to the reduction of fat content at mesenteric, perirenal white adipose tissues, or other body mass, similar to other *Lactobacillus* species (22–24).

Furthermore, AM-ZHY1 significantly reduced the expression of lipogenic genes (*PPAR*γ, *SREBP-1c, ACC1*, and *FAS*) and fatty acid transport-related proteins (*CD36, FABP6*) in high-fat diet-fed zebrafish. However, compared with high-fat diet-fed zebrafish treated with ZHY1, AM-ZHY1 had no significant effect on the expression of *ATGL* and *UCP2* in high-fat diet-fed zebrafish. These results indicated that AM-ZHY1 mainly reduces high-fat diet-induced fat accumulation by reducing fat synthesis and inhibiting fat absorption.

The intestinal barrier function was important as a defense against foreign infections. The permeability and stability of the epithelial barrier maybe dependent on the TJs and adherens junctions between epithelial cells (25). The TJs of the intestinal epithelium were a specialized structure, mainly composed of transmembrane proteins, including *occludin, trillulin* and *claudin* families, and *zonula occludens* (*ZO-1*) (26, 27). These TJ proteins maintained dynamic balance through continuous remodeling and renewal under the strict regulation of extracellular and intracellular factors. The destruction of this dynamic balance would lead to intestinal epithelial barrier dysfunction which would increase intestinal epicellular paracellular permeability, and cause the translocation of luminal toxic substances and bacteria to the interior organization (28). It was reported that, as the TJ expression levels in the intestines of mice with high-fat diet-induced obesity decreased, the permeability of the intestine increased and even caused endotoxemia (29–31). We explored whether AM-ZHY1 had a protective effect on the destruction of intestinal barrier function caused by the high-fat diet. This study had shown that AM-ZHY1 could maintain intestinal health by increasing the expression of a series of TJ proteins (*TJP1a, claudina, claudin7, claudin7b, claudin11a, claudin12*, and *claudin15a*).

The homeostasis of the intestinal microbial community was vital to the gut histologic structure, metabolism, and gut immunity of fish (32, 33). A high-fat diet could cause a bad tilt of intestinal flora and reduce the abundance and diversity of microorganisms (34, 35). In our previous studies, the increase in the abundance of *Fusobacteria* and the decrease in the abundance of *Proteobacteria* indicated that the intestinal health of the zebrafish had improved (36). In this study, the addition of the recombinant bacteria AM-ZH1 could significantly increase the abundance of *Fusobacteria* (*Cetobacterium*) in the guts of high-fat diet zebrafish.

## Conclusion

In conclusion, AM protein was successfully anchored to the cell surface of *L. lactis* ZHY1. This recombinant *L. lactis* AM-ZHY1 could reduce hepatic fat accumulation, relieve liver injury, and inhibit liver inflammation in high-fat diet-fed zebrafish. Furthermore, AM-ZHY1 could maintain the barrier function of the intestinal tract by increasing the expression of intestinal TJ proteins. Moreover, the relative abundance of beneficial commensal bacterium (*Cetobacterium*) was increased in the AM-ZHY1 group.

## Data Availability Statement

The datasets presented in this study can be found in online repositories. The names of the repository/repositories and accession number(s) can be found below: PRJNA739824.

## Ethics Statement

The animal study was reviewed and approved by Feed Research Institute of the Chinese Academy of Agricultural Sciences Animal Care Committee. Written informed consent was obtained from the owners for the participation of their animals in this study.

## Author Contributions

F-LZ, Z-GZ, Y-LY, and ZZ participated in the research design. F-LZ, Z-GZ, and ZZ conducted the experiments and performed the data analysis. F-LZ, Y-YY, RX, C-CG, D-DD, JH, and CR, ZL wrote the manuscript or contributed to the manuscript. All authors contributed to the article and approved the submitted version.

## Funding

This work was supported by grants from the National Natural Science Foundation of China (Grant Nos. 32172958, 3180131599, and 31925038), the earmarked fund for the Modern Agro-industry Technology Research System (SCGWZJ20211104-4), and the Innovation Capability Support Program of Shaanxi (2018TD-021).

## Conflict of Interest

The authors declare that the research was conducted in the absence of any commercial or financial relationships that could be construed as a potential conflict of interest.

## Publisher's Note

All claims expressed in this article are solely those of the authors and do not necessarily represent those of their affiliated organizations, or those of the publisher, the editors and the reviewers. Any product that may be evaluated in this article, or claim that may be made by its manufacturer, is not guaranteed or endorsed by the publisher.
